# Repeatability of Multiparametric Prostate MRI Radiomics Features

**DOI:** 10.1038/s41598-019-45766-z

**Published:** 2019-07-01

**Authors:** Michael Schwier, Joost van Griethuysen, Mark G. Vangel, Steve Pieper, Sharon Peled, Clare Tempany, Hugo J. W. L. Aerts, Ron Kikinis, Fiona M. Fennessy, Andriy Fedorov

**Affiliations:** 1 Brigham and Women’s Hospital, Boston, MA USA; 2 Harvard Medical School, Boston, MA USA; 3 Netherlands Cancer Institute Maastricht University, Amsterdam, Netherlands; 4 Massachusetts General Hospital, Charlestown, MA USA; 5 Isomics, Inc, Cambridge, MA USA; 6 Dana-Farber Cancer Institute, Boston, MA USA; 7 Fraunhofer MEVIS, Bremen, Germany; 8 Mathematics/Computer Science Faculty, University of Bremen, Bremen, Germany

**Keywords:** Diagnostic markers, Computer science

## Abstract

In this study we assessed the repeatability of radiomics features on small prostate tumors using test-retest Multiparametric Magnetic Resonance Imaging (mpMRI). The premise of radiomics is that quantitative image-based features can serve as biomarkers for detecting and characterizing disease. For such biomarkers to be useful, repeatability is a basic requirement, meaning its value must remain stable between two scans, if the conditions remain stable. We investigated repeatability of radiomics features under various preprocessing and extraction configurations including various image normalization schemes, different image pre-filtering, and different bin widths for image discretization. Although we found many radiomics features and preprocessing combinations with high repeatability (Intraclass Correlation Coefficient > 0.85), our results indicate that overall the repeatability is highly sensitive to the processing parameters. Neither image normalization, using a variety of approaches, nor the use of pre-filtering options resulted in consistent improvements in repeatability. We urge caution when interpreting radiomics features and advise paying close attention to the processing configuration details of reported results. Furthermore, we advocate reporting all processing details in radiomics studies and strongly recommend the use of open source implementations.

## Introduction

The field of Radiomics is concerned with the extraction of quantitative imaging features to convert images into a large scale mineable data^1^. Lambin *et al*.^2^ state the Radiomics hypothesis “that advanced image analysis on conventional and novel medical imaging could capture additional information not currently used, and […] that genomic and proteomics patterns can be expressed in terms of macroscopic image-based features.” The prognostic and discriminative power of radiomics features has been explored in cancer imaging with promising results^3–13^ (including tumor locations prostate, lung, head and neck, brain, breast, glioblastoma, etc.).

Prostate cancer is one of the emerging applications with a strong need for improved characterization of the disease using imaging, as is evident from the ongoing efforts to standardize acquisition and reporting of the imaging findings^14,15^. Multiparametric MRI (mpMRI) is a well-established clinical tool used effectively for cancer detection, characterization, treatment planning and response assessment. However, applications of quantitative analysis of mpMRI are very limited in the clinic.

The generally accepted standard of care is to use the Prostate Imaging Reporting and Data System (PI-RADS v2)^15^, which establishes the guidelines for performance of and qualitative interpretation of mpMRI. In the research applications, most of the studies investigating quantitative analysis of mpMRI utilize basic imaging-derived features such as lesion volume^16^, summary statistics of the Apparent Diffusion Coefficient (ADC)^17^ and pharmacokinetic maps estimated from DCE MRI^18^. More recently, early results suggest that radiomics may have a role in differentiating non-cancerous benign prostate tissue from cancer, as well as grading prostate cancer^19^. Fehr *et al*.^4^ combined a set of first and second order texture features computed in ADC and T2-weighted (T2w) in an automatic Gleason pattern classification algorithm. Wibmer *et al*.^5^ also demonstrated that second order texture features on ADC and T2w may differentiate between cancer and normal tissue. They also found a correlation between ADC Entropy and Energy and Gleason score, but no correlation between T2w texture features and Gleason score. In another study Peng *et al*.^6^ found that “[t]he combination of 10th percentile ADC, average ADC, and T2-weighted skewness with CAD is promising in the differentiation of prostate cancer from normal tissue. ADC image features and K^trans^ moderately correlate with GS”. In a very recent development, Bonekamp *et al*. concluded that the mean ADC has performance comparable to that of radiomic-based machine learning in identifying biopsy-confirmed clinically significant lesions^20^. As such, no unequivocal recommendation exists on what, if any, radiomics features can be recommended for PCa characterization.

The premise of radiomics is that quantitative image features can serve as a biomarker characterizing the disease, or allowing prediction of response and thus providing decision support for patient management. To reliably derive conclusions based on any biomarker, a basic requirement is that its value must remain stable between the two measurements, if the conditions remain stable^21–24^. In particular, this means that a biomarker must be stable under usual scanner noise and normal anatomical or physiological deviations. Investigation of biomarker repeatability is a fundamental component of its technical (assay) validation: one of the consensus recommendations for imaging biomarker (IB) validation and qualification in cancer studies^25^ specifically states that “IB precision must be demonstrated early in IB development through single-centre repeatability studies, or few-site reproducibility studies,” which is exactly the aim of our study. We use the the term “repeatability” for this attribute of a radiomics feature (others refer to it as “reproducibility^21,22,26,27^”, or “stability^3,23,24,28^”). Good repeatability is a necessary, but not a sufficient condition for a high predictive power of a feature, meaning that if a feature has a high predictive power, its repeatability must be good. If a feature has a low repeatability, its predictive power must be low, too. But if a feature has a good repeatability, we cannot conclude anything about its predictive power. Gudmundsson *et al*.^24^ demonstrated this aspect in their evaluations of feature importance and stability on different physiological time series.

Considering the repeatability of features is therefore a good measure for pre-selecting features for a classification task, given a large amount of features to select from. Such a pre-selection is necessary since hundreds of feature sets are available for consideration in medical imaging^29^. This number multiplies if we consider different parameters, filters and preprocessing combinations.

Repeatability of imaging biomarkers has been investigated for a number of imaging modalities and applications. Zhao *et al*.^30^ investigated repeatability of manual and computer aided diameter and volume measurements of lung lesions on CT test-retest. Others looked into the repeatability of MRI specific measures like per-voxel ADC^31^ or quantitative parameters maps in T1 and T2*- weighted images^32^. Bologna *et al*.^28^ presented a method for assessing repeatability and predictive power of radiomics features without using test-retest data or multiple segmentations per case. Several studies have investigated a large set of radiomics features on CT lung cancer cases^3,21,22,26,27,33^. All of them found a large number of features with a good repeatability. Zhao *et al*.^21^ found that many features are reproducible even under different CT reconstruction settings, but they also mention that repeatability of texture features is particularly susceptible to varying pre-processing schemes. Hu *et al*.^34^ report 252 of 775 texture features with high repeatability on CT rectal cancer cases and that the influence of various filters on texture features is small. Another set of repeatability studies was conducted on non-small cell lung cancer cases^23,35,36^. While Leijenaar *et al*.^23^ and van Velden *et al*.^35^ report overall good repeatability, Desseroit *et al*.^36^ mention a critical aspect: “repeatability […] varied greatly among metrics” and “depended strongly on the quantization step, with different optimal choices for each modality”. Critical issues were also raised by Emaminejad *et al*.^37^ and Chalkidou *et al*.^38^. Emaminejad *et al*.^37^ investigated various factors influencing texture feature calculation (Entropy) and discovered that they introduce substantial variation. Chalkidou *et al*.^38^ reviewed 15 studies and “found insufficient evidence to support a relationship between PET or CT texture features and patient survival”.

Based on these results, we feel that further emphasis on repeatability is needed in the radiomics literature. At the same time, radiomics analysis is fraught with complexities in identifying the optimal analysis parameters. As an example, we did not identify a consistent recommendation on how pre-filtering should be performed in PCa MRI radiomics characterization^4–6^. Depending on the specific study, image normalization (scaling and shifting) was applied only for texture features^5^, for all features^4^, or not used at all^6^. 3D computation of texture features is only mentioned in one study^5^, while others^4,6^ do not specify whether their computations were done in 2D or 3D. Overall, the description of the preprocessing often lacks details to allow for exact reproduction of the calculations.

Furthermore, most of the existing studies investigating radiomics feature repeatability focus on features extracted from CT. Radiomics analysis of MRI data poses significant challenges due to lack of signal normalization, more common acquisition artifacts, and lower spatial resolution. In their comprehensive review radiomics paper of 2016 Yip *et al*.^39^ state that “the repeatability of MR-based radiomic features has not been investigated” and that “[u]nderstanding the stability of MR-based radiomic features between test and re-test scans can help identifying reliable features for radiomic applications, and thus would be a valuable future study”. We still observe this gap in the present understanding of the feature repeatability applied to MRI analysis in general, and in prostate cancer imaging specifically.

In this study we assess the repeatability of radiomics features using a publicly available dataset^40^ of small prostate tumors in multiparametric prostate MR images (mpMRI). We consider all features implemented in the open source *pyradiomics* package^41^, which are, for the most part, implemented according to consensus definitions of the Imaging Biomarkers Standardization Initiative (IBSI)^29^. We investigate various factors likely to influence the repeatability of features, such as image normalization, 2D/3D texture computation, discretization with different bin widths, and image pre-filtering. Furthermore, repeatability also depends on the accuracy of tumor segmentation at both time points but we did not fully investigate the impact of that factor in this study. In our reporting we focus on disclosing all configuration details and make our implementation and data available. We note that we do not report results for all of the observations (all combinations of image types, regions, choices of normalization, pre-filtering and feature sets). Instead, our goal is to summarize findings of most relevance. This study is an extension of our previous work, where we evaluated the repeatability of volume and apparent diffusion coefficient (ADC)^42^. Those basic imaging features are widely recognized as valuable markers of prostate cancer^16,17,43^.

## Methods

### Image data and segmentations

**Figure 1:**
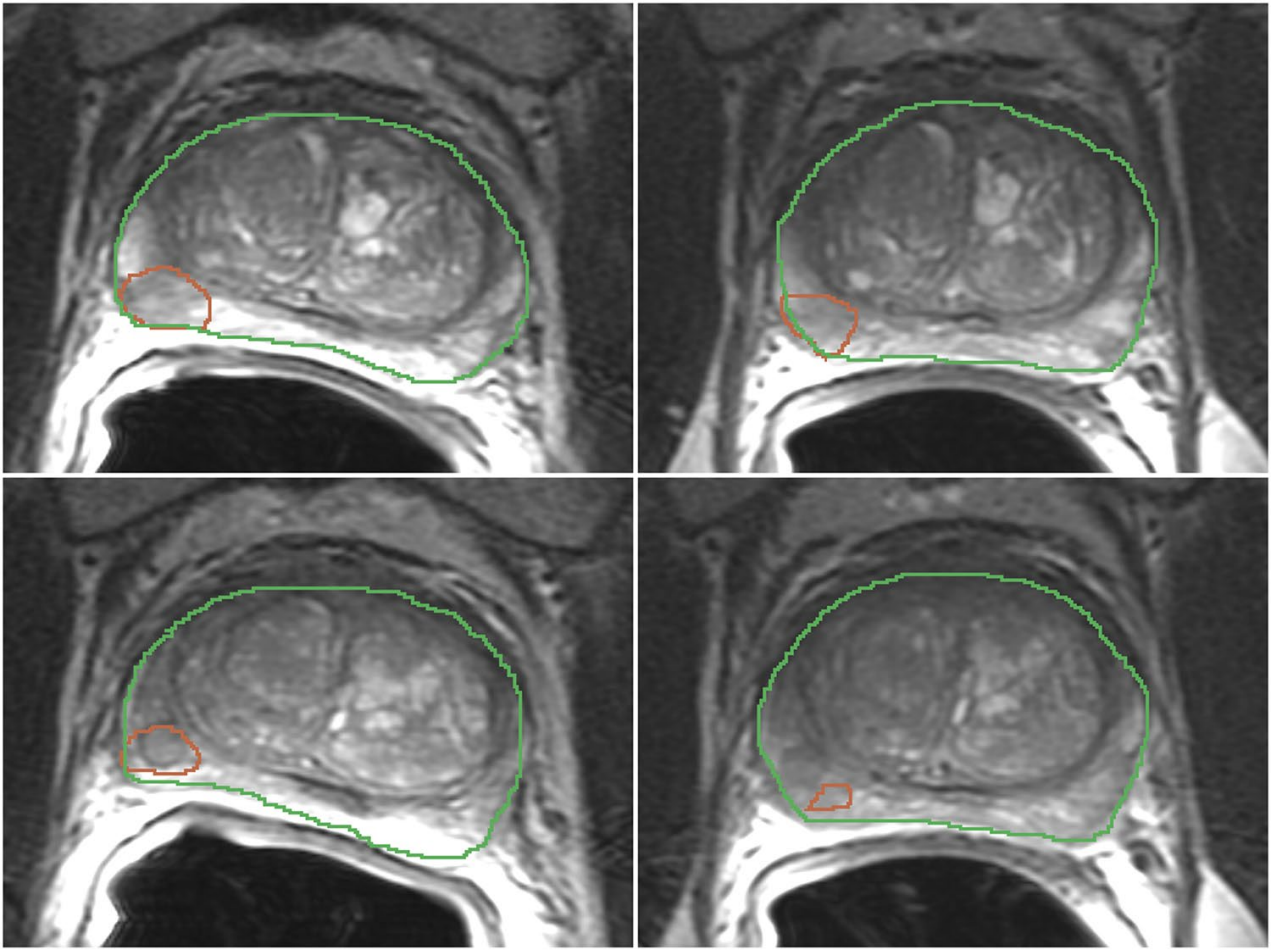
Segmentation of the Tumor ROI (red) and Whole Gland (green) in T2w images for case 7 baseline (left) and follow-up (right) scans. Each slice pair was selected to match location. Top and bottom show consecutive slices going through the same tumor. The individual time points were segmented manually by the domain expert blinded to the other time point. Upon the review of the images corresponding to the time points side by side, it becomes apparent that the location of the segmented region is not consistent for the slice shown in the bottom row.

This study used a previously published, publicly available prostate mpMRI test-retest dataset^40^ composed of fifteen treatment-naïve men with biopsy-confirmed (n = 11) (using a sextant biopsy technique) or suspected (n = 4) prostate cancer (PCa). After providing informed consent, all 15 patients underwent a second MR within two weeks after the first MR, without any interim treatment^42^. From the MRI exam we used the T2-weighted axial (T2w) images (TR 3350–5109 ms, TE 84–107 ms, FOV 140–200 mm), and the Apparent Diffusion Coefficient (ADC) maps derived from Diffusion-weighted MRI (b = 1400 s/mm^2^, TR 2500–8150 ms, TE 76–80 ms, FOV 160–280 mm). See Fig. 1 for an illustration of one of the images used in this study.

A radiologist with 10+ years of experience in prostate mpMRI reviewed all of the images for the individual MR studies using 3D Slicer software (http://slicer.org)^44^. Regions of interest (ROIs) annotated included the suspected tumor, entire peripheral zone of the prostate gland, and the entire prostate gland identified in the baseline and follow-up T2w and ADC images. Image annotation utilized a visualization protocol whereas all of the individual images for a given patient and time point combination were shown to the reader for a single time point. However, the individual timepoints were randomized so that while annotating a given study the reader was blinded to the other time point for the same patient. Notably, all resulting tumor ROIs used for calculating the features were smaller than 0.8 ml. Details relating to the acquisition and annotation of the dataset are described elsewhere^40,42^.

Upon review of the data, one of the subjects (case 1) was excluded from the analysis of ADC features, since one time point was deemed of poor image quality due to noise and observed distortions. As a result, analysis of repeatability of the radiomics features for the ADC images was conducted using the data from the remaining 14 subjects.

Stability of the features is affected by the consistency of the segmentation of the region of interest between the baseline and repeat scans. In absence of the ground truth, we cannot evaluate the absolute accuracy of the manual segmentation performed by the radiologist.

### Feature extraction

Features were extracted for all ROIs using *pyradiomics*, presented earlier in^41^. We extracted features from five feature classes: First Order, Shape, Gray Level Co-occurrence Matrix (GLCM), Gray Level Size Zone Matrix (GLSZM), and Gray Level Run Length Matrix (GLRLM) features (throughout the text, whenever they correspond to implementations in *pyradiomics*, feature classes are capitalized, and feature names as well as preprocessing filters are capitalized and denoted in italics). All features of each of these classes were extracted with the following exceptions: *Compactness1* and *Compactness2*, as well as *SphericalDisproportion* from Shape features were excluded because they are directly correlated to *Sphericity* (based on the definition of the feature, as discussed in the documentation of *pyradiomics*). *Flatness* and *LeastAxis* from Shape features were excluded because some tumor ROIs were only defined on one slice and these features do not yield useful values for non-3D objects. *SumAverage* was excluded because it is directly correlated with *JointAverage*. *Homogeneity1* and *Homogeneity2* were disabled because they are directly correlated to *InverseDifferenceMoment*.

*Pyradiomics* allows preprocessing of (applying filtering to) the original image before feature extraction and offers the following options^41^: *Original* - leave the image unchanged, *LoG* - apply a Laplacian of Gaussian filter, *Wavelet* - apply wavelet filters (all combinations of high- and low-pass filters on each image dimension), *Square* - ${(imageintensity)}^{2}$, *Square Root* - $\sqrt{|image\,intensity|}$, *Logarithm* - $log(|image\,intensity|+1)$, and *Exponential* - ${e}^{(imageintensity)}$. The last four filters also scale the values back to the original image range and restore negative sign if original was negative. For the *LoG* preprocessing we choose kernel sizes (sigmas) 1.0, 2.0, 3.0, 4.0, and 5.0 mm. Each feature was computed with each of the above mentioned preprocessing steps separately. We note that image pre-processing prior to feature calculation is currently not covered by the IBSI radiomics standardization initiative^29^. Here we consider all standard pre-processing approaches implemented in *pyradiomics*, which include pre-processing filters (i.e., *Wavelet* and *LoG*) that have been shown to result in highly predictive feature sets^3,45^.

Bias correction was applied to all T2w images to compensate for intensity non-uniformities using N4 Bias Correction approach^46^ implemented in 3D Slicer^44^.

MR image intensity is usually relative and not directly comparable between images. To reduce this effect we applied intensity normalization. To test the effect of normalization on feature repeatability we included features computed with and without normalization. Whole-image normalization was performed by scaling and shifting the values of the whole image to a mean signal value of 300 and a standard deviation of 100. This means we would expect most of the values in the range of 0–600, assuming normal distribution of the intensities within the image. We also considered normalization based on a biologically comparable reference tissue region, which is assumed to be stable across time points and patients. For this we selected an ROI in a muscle region. The utility of normalization to the muscle reference region has been demonstrate before in prostate imaging studies (e.g., see Huang *et al*.^47^). Compared to the PZ tissue, we expect the muscle region to be more homogeneous, which we believe makes the muscle-reference normalization more robust. The shifting and scaling factor for all image voxels was determined such that the mean signal in the reference ROI changed to 100 and the standard deviation to 10. The smaller range and lower mean for the muscle-reference normalization is due to the fact that the reference region represents only a small portion of the whole image intensity range and that muscle tissue has low intensity. This way the intensities of the whole image maintain a reasonable range after muscle-reference normalization.

Computation of texture features requires discretization (binning) of the image intensities into a limited number of grey levels. This can be done using either fixed number of bins, or the fixed bin size (see section 2.7 of the IBSI guidelines v6^29^). We used the latter discretization approach, as implemented in *pyradiomics*. Leijenaar *et al*.^48^ argue why it is imperative to use a fixed bin width and not a fixed number of bins for discretisation. Additionally, according to Tixier *et al*.^49^ the total number of bins for texture feature computation should be between 8 and 128. Considering our value range of 0–600 after whole-image normalization, we should select bin widths not smaller than 5. However, for non-normalized images as well as muscle-reference normalization the intensity ranges vary and can be much larger. Hence, we selected bin widths 10, 15, 20, and 40 for performing our experiments, which should result in the number of bins below 128 for intensity ranges of up to 5120. We note that image binning as implemented in *pyradiomics* is based only on the intensities that are within the region of interest.

Texture features are computed as various statistics over specific matrices (e.g., co-occurrence matrix for GLCM). The dimensionality of the texture matrix defines the neighborhood (2D vs 3D) over which feature calculation is performed (see the *pyradiomics* documentation for more details). Since the choice between 2D- and 3D-based calculation of texture features is not obvious, and no comparisons of the two were done before, we included the comparison of stability of the two approaches in our study.

Default settings were used for all other configuration parameters of *pyradiomics* feature extraction (see http://pyradiomics.readthedocs.io for further information).

### Measure of repeatability

As a measure of repeatability we report the intraclass correlation coefficient ICC(1,1)^50^. All scans were acquired on the same scanner for the baseline and repeat images, and were annotated by the same radiologist. Annotation was done by a single radiologist completely blinded with respect to subject. Therefore, the variability due to annotation is part of the within-subject variability. The two sources of variability included in the ICC are between-subject and between-scans within the subject. Since there is no systematic difference between the first and second scans, we model both between-subject and within-subject sources of variability as independent random effects and ICC(1,1) is therefore appropriate. The ICC considers the variation between repeated scans on the same subject in relation to the total variability in the population^51^. For our test-retest scenario with two time points it is defined as follows:

$$ICC(1,1)=\frac{BMS-WMS}{BMS+WMS},$$

where BMS is the between-subjects mean squares and WMS the within-subjects mean square^50^. Hence BMS is an estimate for the variance between patients in our study and WMS an estimate for the variance over repeated measurements on the same patient.

The ICC is invariant with respect to linear scaling and shifting. This is a necessary property for using it to compare repeatability of features which operate in different unit and scale spaces. Since fixed thresholds for interpreting the ICC are problematic (see our Discussion section and e.g. Raunig *et al*.^51^), ICCs of different radiomics features should be compared to a reference within the study population. We use the *Volume* ICC as such reference. Tumor volume is an important quantitative measure characterizing PCa, which has been investigated earlier^16^, and evaluation of its repeatability in this specific dataset has already been presented in our earlier work^42^.

### Evaluation approach

We start by evaluating the repeatability of a small subset of features, which were shown by others to perform well in PCa mpMRI radiomics-style analyses^4–6^. Specifically, this initial step of the evaluation focused on the following Intensity and GLCM radiomics features: *Volume*, *Entropy*, *Energy*, *Idm* (Inverse Difference Moment), *Correlation*, *Contrast*, *Variance*, *Skewness*, *Median*, *Mean*, *Kurtosis*, and *10Percentile* (10th percentile of intensity distribution). Using this relatively small dataset, we first explore the effects of whole-image normalization and intensity bin size on the repeatability of those features for image types ADC and T2w. Based on the observations on this reduced feature set, we aim to identify preprocessing and feature extraction parameters that lead to improved repeatability, and continue with the evaluation of the complete radiomics feature set using the selected processing options. Since it is challenging to look into hundreds of features individually, in this phase we focus either on summarizing statistics over all features, or on a selection of the top 3 best performing features per feature group.

The large amount of data generated by our extraction also does not allow us to explore all aspects in detail in this paper. Further details can be found in an extended preprint^52^. The preprint also covers additional analyses not considered in this paper, such as an investigation of registration to mitigate the effect of potentially inconsistent segmentations between timepoints as well as an evaluation of repeatability on Subtraction images (pixel-wise difference between the early post-contrast image phase and the pre-contrast phase of the Dynamic Contrast Enhanced MRI acquisition). Furthermore, the source code accompanying this manuscript includes additional investigations.

### Ethical approval and informed consent

The analysis conducted in this study was performed on the publicly available de-identified human subject data released earlier within the QIN-PROSTATE-Repeatability collection on The Cancer Imaging Archive (TCIA)^40^. Since the data is de-identified, was available prior to the presented study, and no identifiable information was used in the present analysis, our study qualifies for Exemption 4 under the “Basic HHS Policy for Protection of Human Research Subjects”, see https://humansubjects.nih.gov/human-specimens-cell-lines-data.

## Results

To investigate the repeatability of radiomics features for small prostate tumors in mpMRI we analyzed a large set of features under various preprocessing combinations. As there are numerous combinations of image types, regions, choices of normalization, pre-filtering and feature sets, we discuss the most interesting and applicable findings in our results. Additional results can be found in the extended preprint^52^ of this paper.

### Selected features on whole-image-normalized vs non-normalized images

**Figure 2:**
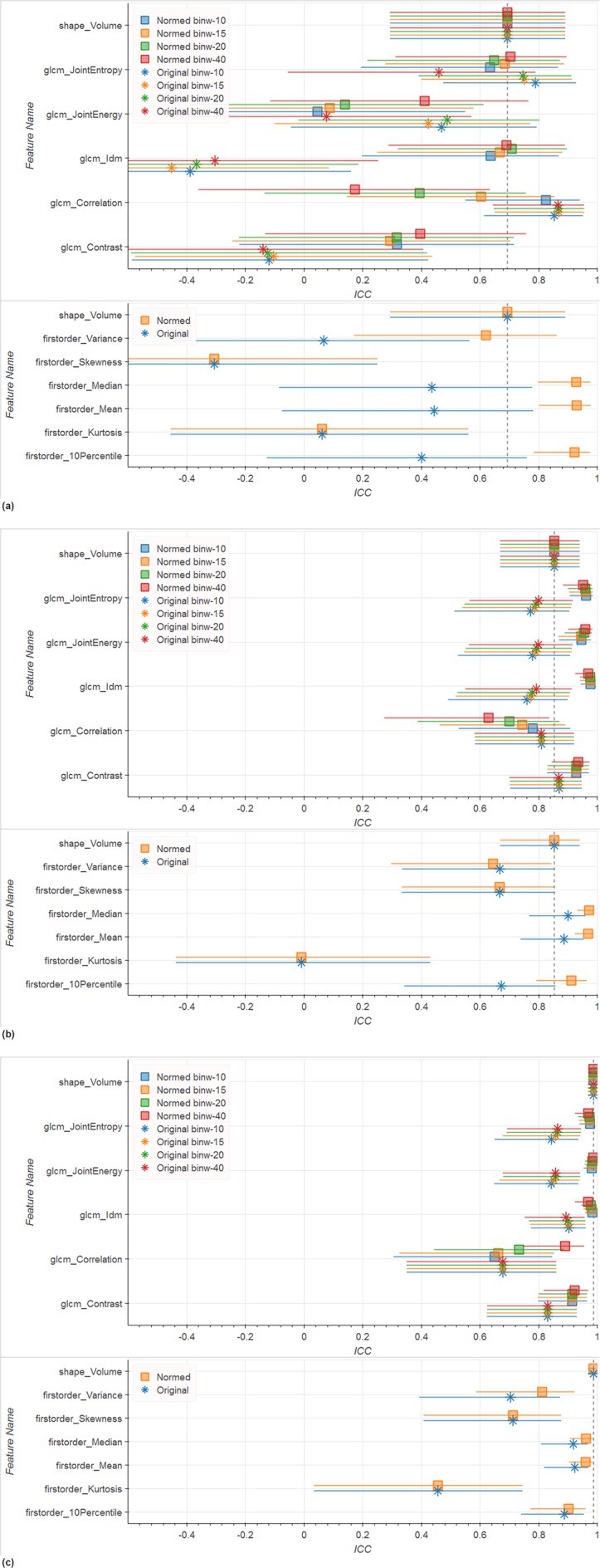
ICC and corresponding 0.9 confidence interval for selected features computed on different ROIs on ADC images. Texture features are computed in 2D. Colors represent the bin width for the texture computations, glyph shape represents if the image was whole-image-normalized or not normalized. No filtering was applied to the image. The dashed line indicates the reference *Volume* ICC. Results show that whole-image normalization tends to improve ICCs while bin width has only marginal influence. (**a**) Tumor ROI (top: texture features, bottom: first order features); (**b**) Peripheral Zone (top: texture features, bottom: first order features); (**c**) Whole Gland (top: texture features, bottom: first order features).

Our evaluation of a small subset of features that were shown by others to perform well in PCa mpMRI analysis yielded the following results. For the Tumor ROI in whole-image-normalized ADC images (see Fig. 2a) *Entropy*, *Idm* (Inverse Difference Moment), *Correlation*, *Median*, *Mean*, and *10Percentile* (10th percentile of the intensity distribution) reach ICCs equal or better than *Volume* (ADC *Volume* ICC = 0.7). In the Peripheral Zone in whole-image-normalized ADC images (see Fig. 2b) *Entropy*, *Energy*, *Idm*, *Contrast*, *Median*, *Mean*, and *10Percentile* reach ICCs around 0.91 or higher, performing better than Volume (ADC *Volume* ICC = 0.85). Looking at the ADC ICCs in the Whole Gland (see Fig. 2c) we observe that no feature reaches the 0.99 ICC of *Volume*. Whole-image normalization leads to improved ICC in ADC images in most cases (see Fig. 2a–c). Notable exceptions are *Variance* in the Peripheral Zone as well as *Entropy*, *Energy*, and *Correlation* in the Tumor ROI. For these exceptions the difference to the ICC of the corresponding whole-image-normalized feature was always smaller than 0.1. Note that *Skewness* (measure of asymmetry of the distribution about the mean) and *Kurtosis* (measure of peakedness of the distribution) are by definition not influenced by whole-image normalization. However, they also never reach the reference *Volume* ICC.

**Figure 3:**
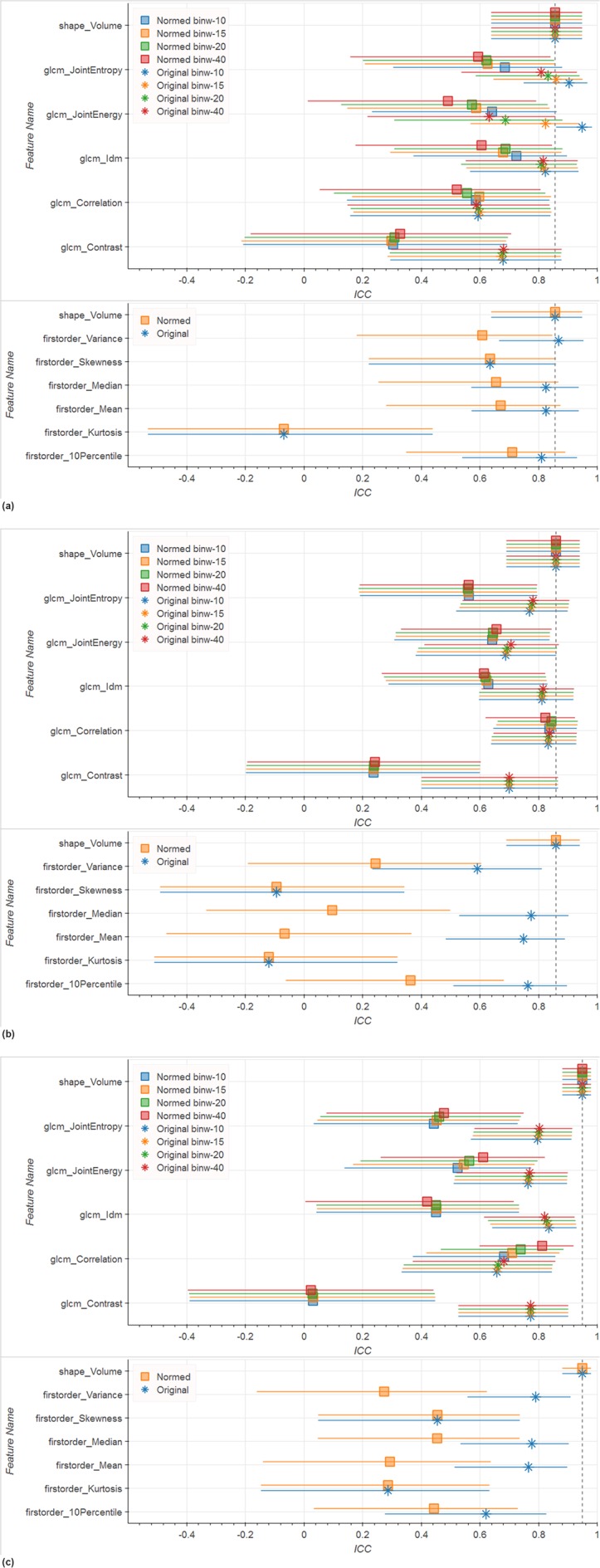
ICC and corresponding 0.9 confidence interval for selected features computed on different ROIs on T2w images. Texture features are computed in 2D. Colors represent the bin width for the texture computations, glyph shape represents if the image was whole-image-normalized or not normalized. No filtering was applied to the image. The dashed line indicates the reference *Volume* ICC. Results show that omitting whole-image normalization tends to improve ICCs while bin width has only marginal influence. (**a**) Tumor ROI (top: texture features, bottom: first order features); (**b**) Peripheral Zone (top: texture features, bottom: first order features); (**c**) Whole Gland (top: texture features, bottom: first order features).

On T2w images neither in the Peripheral Zone, nor in the Whole Gland does an ICC of any feature reach the *Volume* ICC (see Fig. 3b,c), which is 0.86 for the Peripheral Zone and 0.95 for the Whole Gland. Only in the Tumor ROI *Entropy*, *Energy*, and *Variance* reach an ICC higher than the reference T2w Volume ICC of 0.86 (see Fig. 3c). Contrary to ADC images, whole-image normalization leads to lower ICCs in T2w images. The only exception is *Correlation*, which has a higher ICC for the Whole Gland when normalized.

In the following we report further results based on whole-image-normalized ADC and non-normalized T2w images only, since results in this section showed better overall repeatability under these configurations.

### Influence of different bin-widths

**Figure 4:**
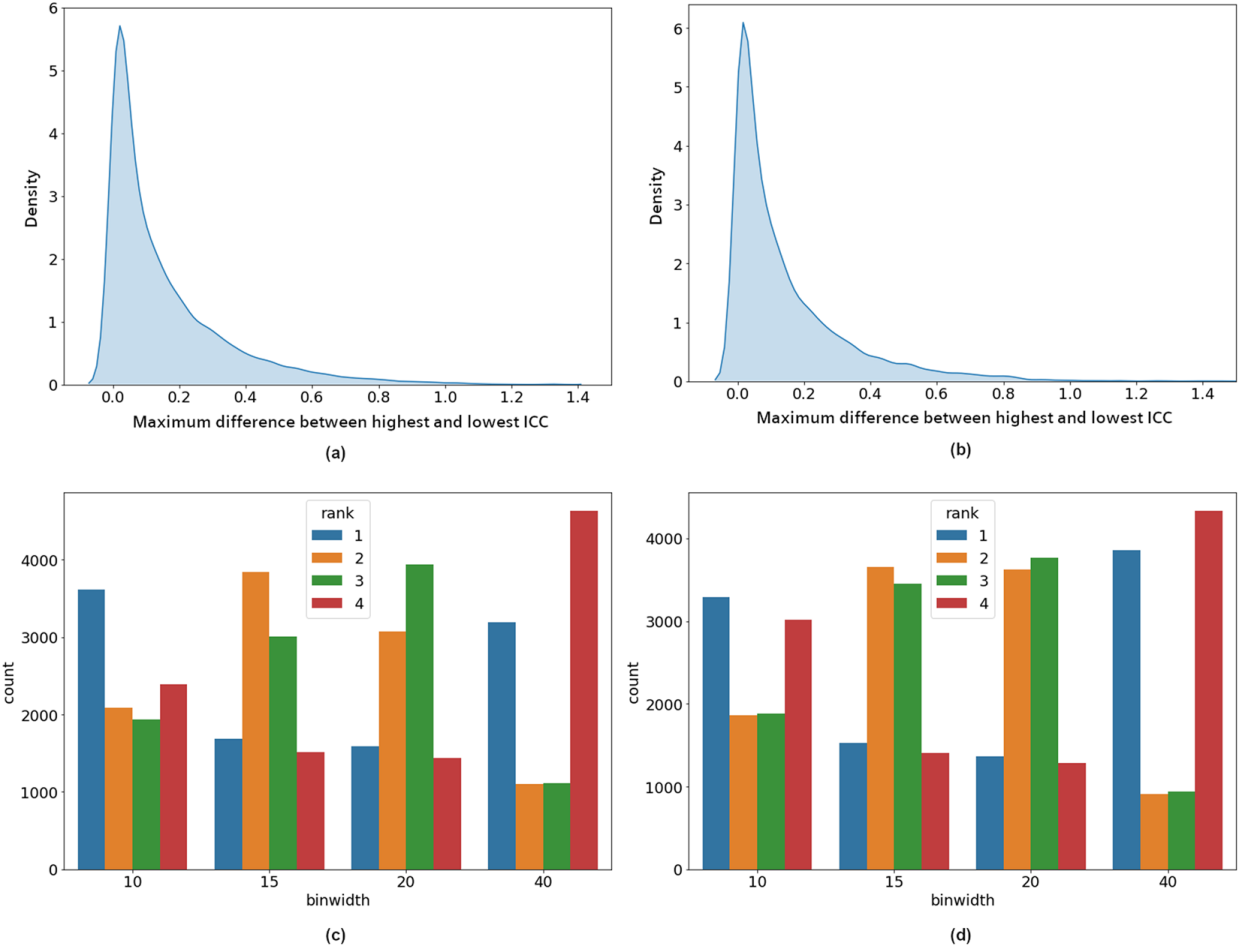
(**a,b**) Kernel density estimation plots for the ICC range over four bin widths, for all combinations of texture features and pre-filtering options on (**a**) ADC and (**b**) T2w images. For most feature and pre-filtering combinations, this range is small. (**c,d**) Histograms of the ICC ranks for all texture feature and pre-filter combinations for each bin width on (**c**) ADC and (**d**) T2w images. Bin widths 10 and 40 are very often associated with the best ICCs but similarly often with the worst ICCs. Bin widths 15 and 20 are more often associated with ICCs in the middle ranks. Hence bin widths 15 or 20 are a better choice to estimate the representative average repeatability of features.

Figures 2 and 3 indicate that different bin widths do not result in very strong variations of the ICC for most features of the selected subset. The kernel density estimation (KDE)^53,54^ plot in Fig. 4a,b support this observation. It shows the distribution of the maximum difference between highest and lowest ICC per feature depending on bin width for all GLCM features and all pre-filtering options. For the majority of features the maximum difference is around 0.2 or lower.

Another insight into the influence of bin width can be gleaned from Fig. 4c,d. We ranked the ICC for each feature depending on the bin width and plotted the rank distribution. We can see that for the lowest and highest bin widths, the best and worst ranks are appearing about equally often. Bin width 15 and 20 cover the middle ranks.

In the following we report all results based on bin width 15, since the results in this section indicate that this yields a reasonable average estimate of the feature repeatability.

### Top 3 features per feature group

**Figure 5:**
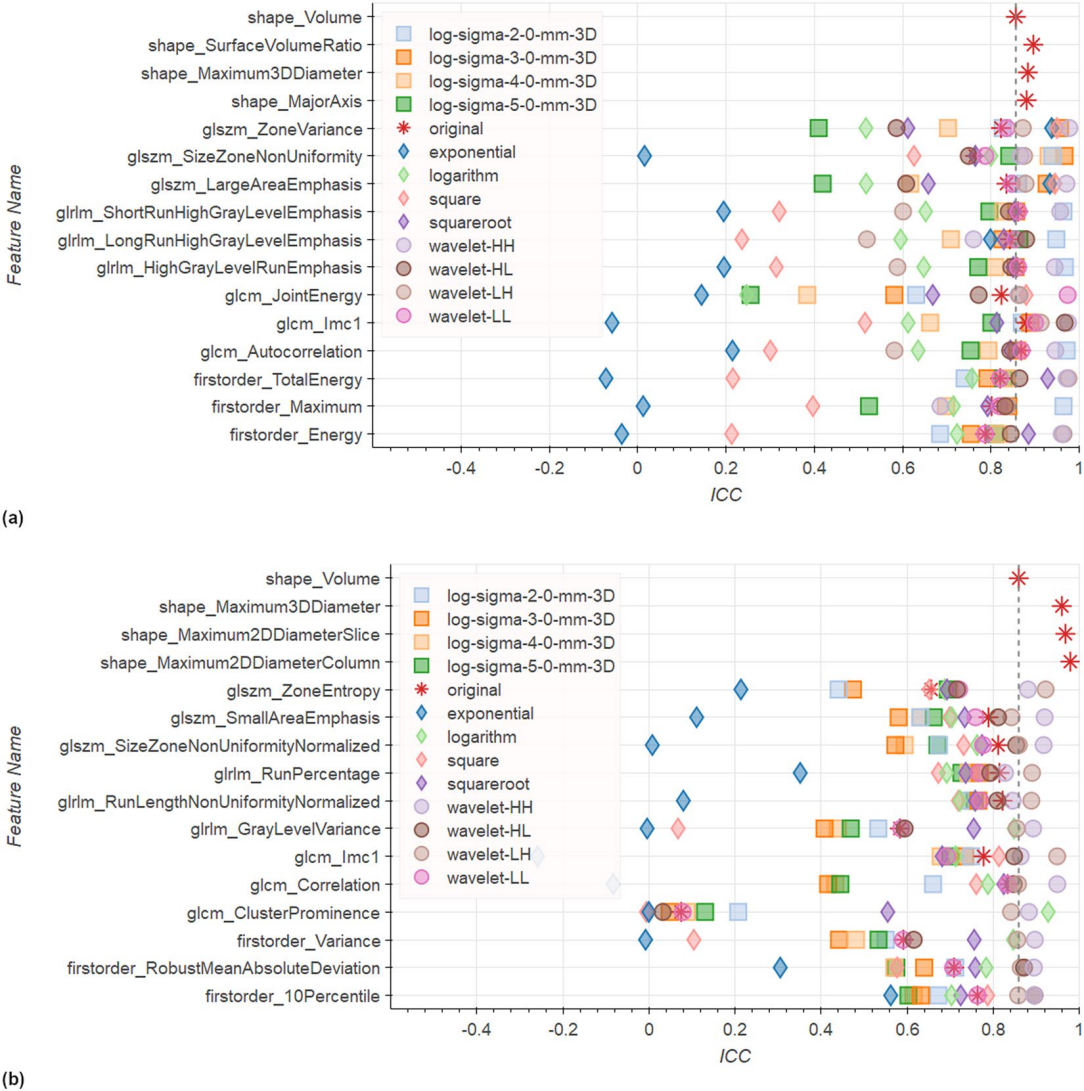
Top 3 features for each feature group by ICC in T2w images for (**a**) the Tumor ROI and (**b**) the Peripheral Zone. Results in these figures illustrate that ICCs are spread over a wide range depending. Also some filters have a consistently low performance. However, no filter consistently performs above reference. We can also see that some shape filters have a higher repeatability than *Volume*. See Supplementary Dataset 1 for confidence intervals for the ICCs in this figure.

Given all possible combinations of pre-filtering options, we selected the 3 most repeatable features for each of the feature classes implemented in *pyradiomics* (namely Shape, First Order, GLCM, GLSZM and GLRLM). We then investigated whether any specific pre-filtering approach consistently resulted in improved repeatability of these selected features. For these top 3 features Fig. 5 illustrates the range of ICCs under all pre-filtering options for the Tumor ROI and Peripheral Zone in T2w images. We observe that other shape features have a better repeatability than Volume. They also, by definition, are not influenced by any pre-filtering. The ICCs of other features are scattered over a wide range. In the Tumor ROI (Fig. 5a) no single filter appears to result in consistently more stable features. However, several filters consistently result in ICCs below Volume ICC (e.g., *Logarithm* and *Exponential*). In the Peripheral Zone, only a few *Wavelet* filters yield ICCs above the reference for all top 3 features with the exception of GLCM *ClusterProminence*, for which also *Logarithm* filtering reaches a higher ICC. On the low end particularly the *Exponential* filter performs consistently weak.

**Figure 6:**
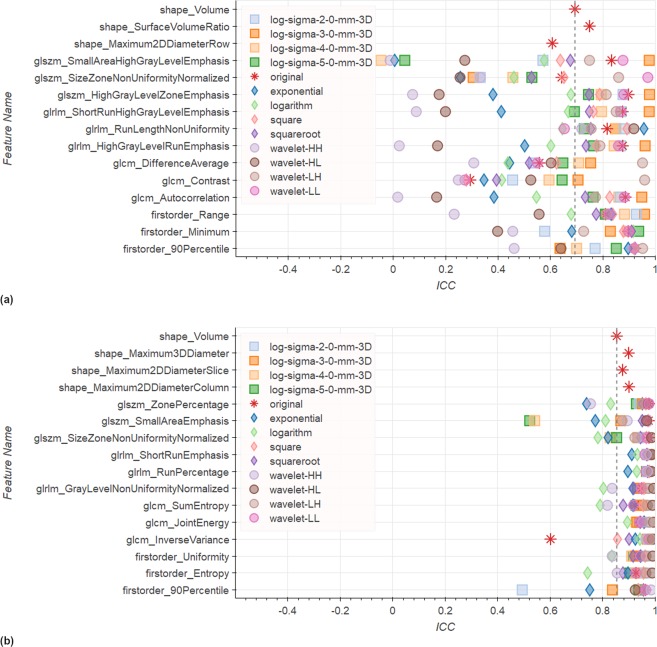
Top 3 features for each feature group by ICC in ADC images for (**a**) the Tumor ROI and (**b**) the Peripheral Zone. Results in these figures show again a wide spread of ICCs in the Tumor ROI, with some filters having tendency towards high ICCs, however, not consistently. For the Peripheral Zone many filters reach consistently above reference repeatability. Overall, ICCs are also much less spread. See Supplementary Dataset 1 for confidence intervals for the ICCs in this figure.

Figure 6 illustrates the same top 3 analysis on ADC images for Tumor ROI and Peripheral Zone. In the Tumor ROI the ICCs are scattered over a wide range again (see Fig. 6a). Even though many filters are related to high ICCs on several features, no consistent trend can be observed. A few features have a strong tendency towards yielding high ICCs, like *LoG* sigma 3.0 mm, but we can always find an exception. In the Peripheral Zone most filters are associated with ICCs above the reference for almost all features (see Fig. 6b). Also, we observe that the spread of ICCs depending on the pre-filter is much smaller with a few exceptions (e.g., GLSZM *SmallAreaEmphasis*, or Firstorder *90Percentile*).

### Overview of pre-filter performance over all features

**Figure 7:**
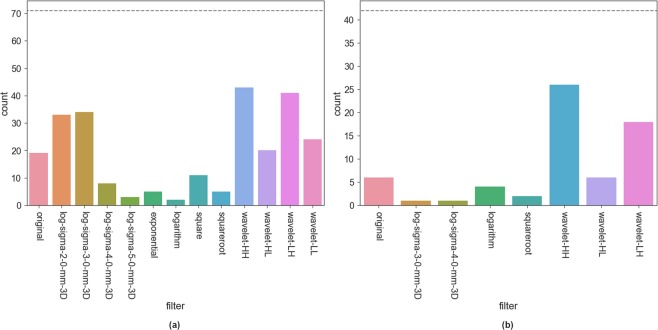
Overview of how often the particular pre-filters appear among the features which reach an ICC higher than Volume on T2w images for (**a**) Tumor ROI and (**b**) Peripheral Zone. Dashed line indicates total number of features with an ICC higher than *Volume*. Note that for one feature several filters can appear. Results in these figures illustrate that some filters are consistently more often related to high repeatability than others. However, no filter comes even close to being always related to high repeatability.

**Figure 8:**
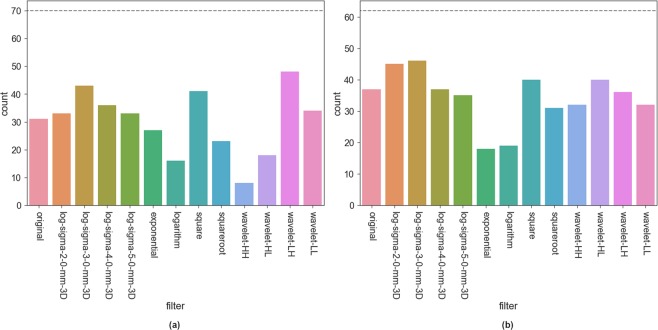
Overview of how often the particular pre-filters appear among the features which reach an ICC higher than Volume on ADC images for (**a**) Tumor ROI and (**b**) Peripheral Zone. Dashed line indicates total number of features with an ICC higher than *Volume*. Note that for one feature several filters can appear. Results in these figures illustrate that almost all filters are related to high repeatability in about half of the above-reference group. However, no filter comes close to being always related to high repeatability.

For an overview of the influence of pre-filtering options on repeatability of all features we considered all features and pre-filter combinations on T2w and ADC images in the Tumor ROI and Peripheral Zone. We selected all of those combinations that had ICC above the corresponding Volume ICC. The plots in Figs 7 and 8 show how often a particular filter appears among those for Tumor ROI and Peripheral Zone in T2w and ADC images. On T2w Tumor ROI *Wavelet-HH*, *Wavelet-LH*, and smaller sigma *LoG* filters are most prominent. Also the other *Wavelet* filters as well as the original image (no filter applied) often lead to high ICC values. On T2w Peripheral Zone only *Wavelet-HH*, and *Wavelet-LH* stand out. In the Tumor ROI on ADC images *Original* and *LoG* filters perform well, while among the *Wavelet* filters *Wavelet-LH* and *Wavelet-LL* stand out. Among the Single Pixel filters *Square* shows a strong performance. For ADC Peripheral Zone the filters corresponding to high ICCs are more equally distributed. The only exceptions are *Exponential* and *Logarithm*, which both are less often associated with good repeatability. Overall, no filter is consistently associated with high ICC values - neither on T2w nor on ADC images (see Figs 7 and 8 for details).

### Normalization against a muscle reference region

**Figure 9:**
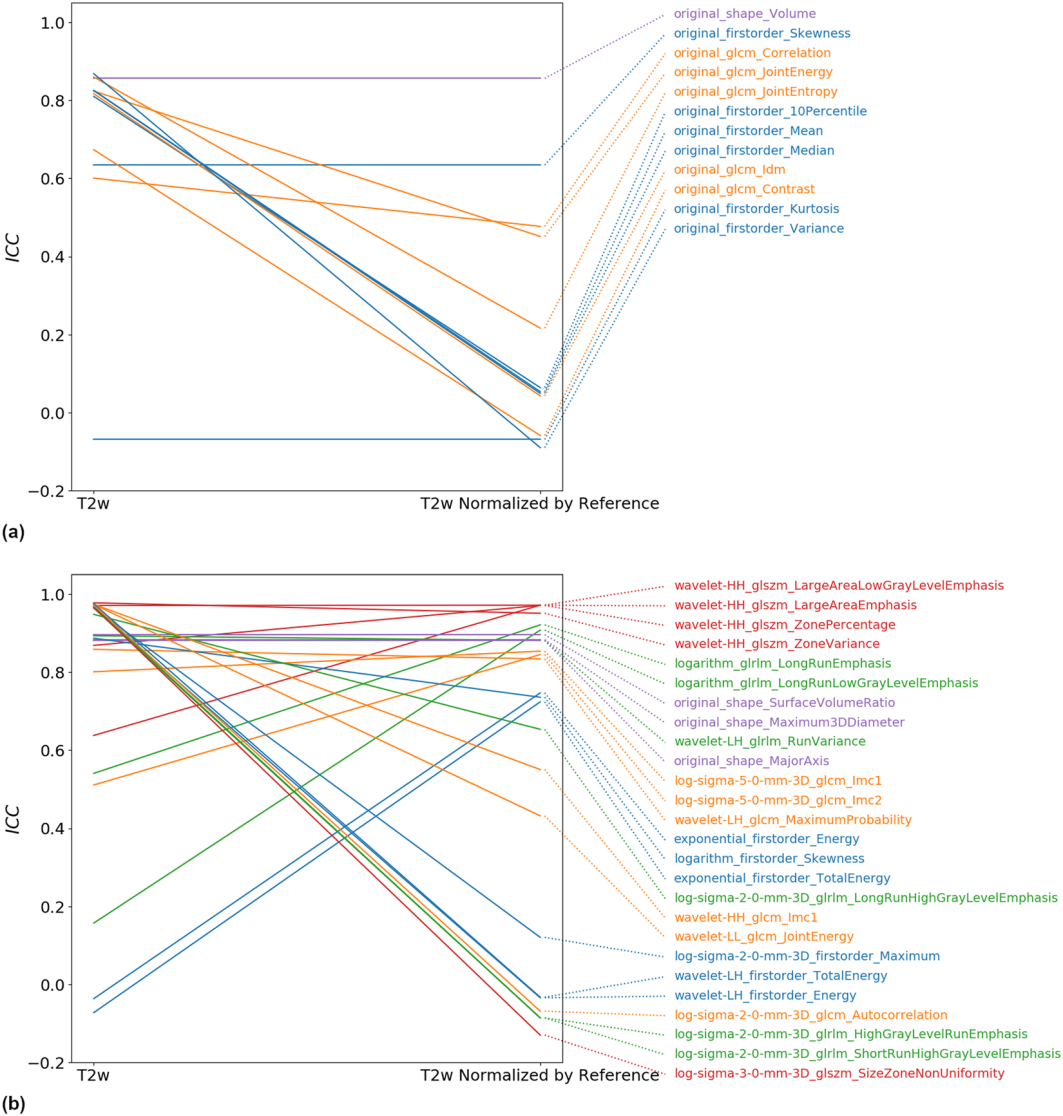
Change in Tumor ROI ICCs from T2w to T2w normalized by a muscle reference region for (**a**) literature recommended features, (**b**) 3 most stable features from each of the feature classes. Note that for each image configuration in (**b**) the top 3 features were selected (hence up to 6 features per feature group are plotted). Results in this figure show that for many features, which have a high repeatability without normalization, normalization by a muscle reference region decreases repeatability. For some features, however, muscle-reference normalization improves repeatability.

Normalization against a muscle reference region on T2w results in a strong decline of repeatability for most of the selected features (see Fig. 9a). We also again selected the top 3 most repeatable features per feature class for the original T2w as well as the T2w that was normalized against a muscle reference region. Figure 9b shows that some features remain stable but repeatability of most original T2w top 3 features strongly declines on muscle-reference-normalized T2w and vice versa.

## Discussion

Despite the small sample size and iterative exploratory approach to conducting the evaluation, our study of radiomics feature repeatability resulted in a large number of observations. To a degree, this was caused by our attempts to find patterns and to be able to explain the results and make specific recommendations. In the following we discuss our interpretation of the main findings of the study. Additional findings are reported in our preprint^52^ of this report, including further preprocessing options as well as an analysis of Subtraction images (SUB). We excluded SUB images from this study, because results did not provide additional insights. Furthermore, they are derived images, obtained by subtracting time frames of the DCE acquisition, hence introducing another source of uncertainty (frames to be subtracted are selected manually, there is a possibility of motion between the time frames). Finally DCE provides limited complementary value in prostate mpMRI^55^.

In this study we focus on repeatability under the premise of a realistic clinical patient re-evaluation, i.e., patients returning for a follow-up scan. Hence differences in patient position as well as the state and layout of organs (e.g., rectal/bladder filling) are expected. A radiomics feature useful for clinical practice must be robust to such deviations. Another type of repeatability study would be to consider consecutive scans of the same patient without repositioning. This would focus only on repeatability variations induced by the scanner only, and was studied elsewhere (e.g., see the study by Sadinski *et al*.^31^). To the best of our knowledge, there is no publicly available dataset corresponding to the latter design, and therefore repeatability of radiomics features under such setting was not feasible in our study.

Among the features reported in the literature for prostate cancer analysis^4–6^ in T2w and ADC MR images, we observed good repeatability (ICC ≥ Volume ICC) for *JointEntropy*, *Idm*, *Median*, and *Mean*. However, we could not confirm good repeatability of other features calculated over Tumor ROIs. Notable examples include *Kurtosis* and *Contrast*, which in all cases are underperforming compared to the ICC of *Volume*, in some cases reaching values close to 0. Furthermore, even features with good repeatability showed these only under specific preprocessing configurations. On our data, whole-image normalization led to improved ICCs for most features calculated from ADC images, while for T2w images whole-image normalization did not result in ICC improvements.

The observation that whole-image normalization improved repeatability for ADC is not completely unexpected. Although ADC is a quantitative measure that is supposed to be consistent across exames and platforms, variability of around 5% was observed even under perfect conditions for a temperature controlled phantom^56^. Several prominent studies in prostate MRI radiomics, e.g., see Fehr *et al*.^4^ and Kwon *et al*.^57^, and elsewhere^58^ apply normalization while analyzing ADC images. Others, like Bonekamp *et al*.^20^, do not apply normalization to ADC images. In practice, there does not seem to be a consensus on the use of normalization for ADC maps.

A possible explanation of the lack of improvement in T2w features’ reproducibility extracted from the whole-image-normalized images could be the following. One of the main reasons we want to normalize MRI images is because we know different scanners and/or protocols yield different images. In the present dataset though, the same scanner and protocol is used. Therefore, the pre-normalization scans may be more comparable than may be expected in a clinical setting. Coupled with the fact that whole-image normalization uses the entire image to determine the mean and standard deviation, one might argue that in this case, noise over the entire image (e.g., due to different positioning, different bowel filling, etc.) might lower the comparability of intensities in the target region (prostate). The same reasoning might explain the ambiguous results for applying muscle-reference normalization to T2w images, resulting in improvements for some but decline in repeatability for other features.

As we saw in Fig. 4, even when the bin width is selected within the recommended limits^48,49^, it still has an influence on the repeatability, although it is not too strong in most cases. Nevertheless, the differences we observed lead us to advice to evaluate the influence of bin width in any new study.

Our investigation of pre-filtering options and parameterization of the texture feature computation revealed further challenges in extracting repeatable radiomics signatures. We found that the use of pre-filtering introduces even more variation in the ICCs per feature across the various pre-filtering options (see Figs 5 and 6). Notable exception is the relative stability of features in the Peripheral Zone ROI for the ADC images (Fig. 6b). Furthermore, our analysis of how often each filter is related to an ICC above *Volume* reference (Figs 7 and 8) again reveals that we cannot suggest a filter that consistently improves repeatability.

Nevertheless, we can observe certain trends for filters that predominantly relate to below-reference ICCs. On T2w images these are all single pixel filters as well as large sigma *LoG*. On ADC images the results are less consistent. *Logarithm* and *Exponential* have low repeatability in the Peripheral Zone, while for the Tumor ROI *Logarithm*, *Wavelet-HH*, and *Wavelet-HL* have the weakest performance. Hence, based on our results, we recommend to leave these filters out for the designated image types and structures.

However, in any case there are still many other filters which yield high ICCs, but not consistently enough to be able to pick a few for general recommendation. Even if we narrow down to a specific image and structure, we cannot single out a small set of pre-processing configurations which consistently result in improved ICCs. The results are too diverse. Depending on the feature, different preprocessing options yield the best repeatability.

There is an exception, however. Our results show several shape features (e.g., *SurfaceVolumeRatio* and different uni-dimensional diameter measurements) with a better repeatability than *Volume*. By definition these are also invariant under any of the investigated pre-processing options. Furthermore, these shape features are correlated to *Volume* and capture less information about shape than *Volume*. Hence most of these features are not likely to add any information that is not captured by *Volume* already.

Even though we could not find configurations which consistently improved the repeatability for all or most features, we still found many features, which - under certain configurations - have a better repeatability than our reference. For the Tumor ROI about 70 features have a higher ICC than *Volume* on T2w as well as on ADC (see Figs 7 and 8). This could indicate that different features simply require different configurations. However, we don’t believe this is the case, considering the small study size plus the fact that no obvious pattern emerges between certain pre-processing configurations and sub-groups of features. Furthermore, we were not able to fully reproduce the good repeatability of features reported in literature for PCa mpMRI on our dataset. This is another indication that there are many factors - even beyond the ones we assessed in this study - that influence the repeatability of features.

There are multiple factors that affect feature repeatability. An important one is the consistency in identification of the region of interest between the two timepoints. Even though the ROIs used in this study were contoured by an expert radiologist with 15+ years of experience in prostate MRI, we can expect that the segmentations will not be perfectly consistent between time points. Figure 1 illustrates that it is not always clear if the segmented regions really completely represent the same tissue region. However, a perfect match of manually defined ROIs cannot be expected in practice. The potential inconsistencies in region definition reflect the challenges of utilizing radiomics for decision support that might be encountered in clinical practice.

In our study, we made the decision to use ICC as the measure for evaluating repeatability. We use the ICC because it is commonly used in radiomics studies, and as such is a *de facto* convention in the field (e.g., see^3,23,24^). It also has the advantages of being invariant with respect to linear scaling and shifting. Some authors indicate fixed thresholds for interpreting the ICC^23,34^. However, these can only be valid as a reference if our population variance was expected to be comparable to the ones used in other studies. In general, this is not the case though. As Raunig *et al*. note^51^: “ICC values for a very heterogeneous subject sample may yield very [sic] nearly perfect correlation based solely on the between-subject variance”. Hence, ICCs of different radiomics features over the same population of subjects should be compared to a reference within this population. Considering those limitations of the absolute ICC threshold, we use the *Volume* ICC as the reference. Tumor volume is an important quantitative measure characterizing PCa, which has been investigated earlier^16^, and evaluation of its repeatability in this specific dataset has already been presented in our earlier work^42^.

Another measure which is commonly used to assess repeatability is the repeatability coefficient (RC)^51,59^. However, the RC is not invariant with respect to scaling and it is denoted in absolute units of the feature. If two features are not expressing their values in the same units and scale space, it is not valid to compare them based on their RC. Since most radiomics features are abstract measures with no direct real-world interpretation we cannot assume that they operate in the same unit and scales space and thus cannot not use the RC for comparing different features. The RC is rather designated for assessing the agreement of two methods, measuring the same quantity^59^, and exposing the expected absolute differences between repeated measurements (limits of agreement).

Our study has limitations. A number of them are inherent to the dataset we used to extract the features (the limitations of the study that generated the MRI data and annotations are discussed extensively by Fedorov *et al*. in^42^): small sample size, small volumes of the identified tumors, lack of the analysis of multi-reader consistency. Specific to our analysis, we did not consider rigorous statistical modeling and testing for our evaluation in this study for several reasons. First, the sample size is rather small. Nevertheless, since there are no existing studies investigating repeatability of the mpMRI features in the prostate, we argue our results are nevertheless of value for the radiomics community. Second, the intention of this paper is an overview of the effects of pre-processing variations on the repeatability of radiomics features. A thorough statistical analysis of all the variations considered in this paper would extend the scope. Since all data of this study is available, we hope this will encourage researchers to perform rigorous statistical analysis as an extension to this study.

Evaluation of diagnostic performance of the features is out of scope of this manuscript. This is due to the small sample size, large number of features, non-binary nature of characterization of the disease by the radiologist (suspicion characterization was done using the 5-point PI-RADS v2 scale, as discussed in^42^), and lack of targeted biopsy samples allowing to confirm the cancer grade for the annotated tumor regions. We also note that evaluation of repeatability is important on its own, as discussed in^60^. As an example, lung CT RIDER dataset was used to study repeatability of image-derived measures, and did not consider diagnostic performance, see studies by Zhao *et al*.^61,62^. Understanding of the repeatability of the features derived from that dataset has been instrumental for feature selection in numerous radiomics studies, such as^3,63^.

Inherently, our conclusions are specific to the dataset, image preprocessing and feature extraction approach, and the ICC as a measure of repeatability. There is an extensive variety of widely used repeatability measures in statistics, many of which are comprehensively summarized by Barnhart *et al*.^60^, and others proposed by imaging researchers, such as those used by Chirra *et al*.^64^. Our goal, however, was not to perform a comprehensive comparison of repeatability measures, but to keep the scope of this study focused, and utilize a measure that is already broadly accepted in the radiomics community. Similarly, it was not our goal to comprehensively explore the various options for image intensity normalization. Although various approaches to intensity normalization have been proposed (e.g., see Nyul *et al*.^65^), they may be applicable under assumptions that do not hold for our data (e.g., absence of pathology or imaging artifacts^66^). Perhaps more importantly, we stress that the normalization approaches we utilize (bias correction, whole-image normalization, and muscle-reference normalization) are employed in prominent recent studies in prostate MRI radiomics (see Bonekamp *et al*.^20^ and Fehr *et al*.^4^). Investigation of alternative approaches to normalization and agreement assessment are certainly justified, but were deemed outside of the scope of the present study.

## Conclusion

Our study shows that radiomics features, as evaluated on the specific PCa mpMRI dataset we used, vary greatly in their repeatability. Furthermore, repeatability of radiomics features evaluated using ICC is highly susceptible to the processing configuration. Even on our small study population, the results already show that the type of image, preprocessing, and region of interest used to evaluate the feature can vastly change the repeatability of certain features. This could contribute to the explanation of why feature recommendations among recent studies are not consistent, and why we could not confirm good repeatability for some of the literature reported features.

We suggest caution when utilizing prior studies as a basis for pre-selection of radiomics features to be used in radiomics signatures. Whenever possible, repeatability analysis on a representative dataset should be done as part of the study-specific feature selection procedure. The dataset used in our study is publicly available^40^, and can be used to facilitate such feature selection procedures for the study-specific radiomics feature extraction procedures. If repeatability analysis is not possible or is impractical, and prior evidence is used for feature pre-selection, we recommend paying close attention to the reported configuration of the feature extraction process. Furthermore, it is important to consider whether the relevance of the assumptions on the reported study population are also valid for the planned study.

When publishing findings on radiomics studies (be it on repeatability or any other performance measure) we strongly advocate reporting of all the details describing the preprocessing and feature extraction procedures. To increase reproducibility of study findings we also strongly recommend following the consensus definitions of features (such as those proposed by the IBSI initiative)^29^ and making the implementation available. When sharing of the dataset is not possible, we recommend that the pertinent details about the study population are reported to help with the interpretation of conclusions of the study. As one example, it is common that distribution of the tumor volumes is not summarized, although it is widely recognized that the size of the region of interest has a strong effect on the measurements extracted. To support reusability and further investigation, our study utilized a publicly available dataset^40^, and the open source radiomics library *pyradiomics*^41^. The calculated radiomics features and evaluation scripts are also published as open-source. This makes it possible to apply alternative implementations of radiomics feature extraction tools to the same dataset, and compare the result with the radiomics features evaluated in this study.

For the specific dataset and radiomics feature extraction approach considered in this study, we were not able to determine a general set of universally stable feature and preprocessing recommendations. Nevertheless, we found many features with a considerably higher repeatability than our reference (*Volume*). Most prominently these include the 3 top performing features for each feature group. Based on the analysis of our data, these can be strong candidates for inclusion into radiomics signatures. We consider a specific study of the predictive power of these candidates on a different dataset valuable future work.

## Supplementary information

Supplementary Dataset 1

## Data Availability

The MRI datasets analyzed in this study are from the QIN-PROSTATE-Repeatability TCIA collection^40^. The extracted features used for all analyses in this paper are located at: https://github.com/QIICR/QIN-PROSTATE-Repeatability-Radiomics/tree/master/EvalData.

## Code Availability

*Pyradiomics* is available at: https://github.com/Radiomics/pyradiomics (the specific version used in this study is v1.3.0.post59 + g2e6d2c1, available at https://github.com/Radiomics/pyradiomics/tree/2e6d2c1).

Code to perform the analysis and figure generation is available at: https://github.com/QIICR/QIN-PROSTATE-Repeatability-Radiomics.

This repository also contains example code on how the features were extracted from the image data.
